# The Effects of *Nosema apis* and *Nosema ceranae* Infection on Survival and Phenoloxidase Gene Expression in *Galleria mellonella* (Lepidoptera: Galleriidae) Compared to *Apis mellifera*

**DOI:** 10.3390/insects12100953

**Published:** 2021-10-19

**Authors:** Erkay Özgör

**Affiliations:** 1 Department of Molecular Biology and Genetics, Cyprus International University, 99258 Mersin-10, Turkey; eozgor@ciu.edu.tr; Tel.: +90-3926711111; 2 Cyprus Bee and Bee Products Research Centre, Cyprus International University, 99258 Mersin-10, Turkey

**Keywords:** *Nosema apis*, *Nosema ceranae*, *Galleria mellonella*, *Apis mellifera*, infection, phenoloxidase gene expression

## Abstract

**Simple Summary:**

The greater wax moth is known as one of the pests in the beekeeping sector. *Nosema ceranae* and *Nosema apis*, which are the most significant microsporidia for adult honeybees, are present in the greater wax moth grown at laboratory conditions and collected from apiaries. In this study, single *N. apis* and *N. ceranae* infection and mixed infection groups have been created for each insect to detect the colonization capability of *Nosema* species and the effect of microsporidial infection on survival and phenoloxidase gene expression. For this purpose, greater wax moth and honeybee experimental groups were fed with 10^6^ single- and mixed-*Nosema*-spore-containing diet, and survival rates of wax moths and bees were recorded. In addition, changes in the level of expression of the phenoloxidase-related gene, which plays a significant role in invertebrates’ immune reaction, was also observed. Honeybees have some mortality rates in mixed and single *Nosema* infection cases, the absence of death in greater wax moth shows that the greater wax moth exhibits a resistance towards *Nosema* species. While the expression of phenoloxidase gene increased in honeybees, the level of phenoloxidase mRNA did not change in the greater wax moth. This shows that the infection remains stable in the wax moth, unlike honeybees.

**Abstract:**

The study aims to prove the possibility of colonization of *N. apis* and *N. ceranae* to the intestine of the greater wax moth, detect the differences of greater wax moth based on the presence of *Nosema* species and examine the effect of *Nosema* species on the phenoloxidase level of greater wax moth compared with honeybees. Each group was fed on the 1st day of the experiment with its appropriate diet containing 10^6^ *Nosema* spores per insect. Each group was checked daily, and dead insects were counted. Furthermore, changes in the level of expression of the phenoloxidase-related gene after *Nosema* spp. treatment on the 6th, 9th and 12th days, which was detected by Q-PCR, and the mRNA level of phenoloxidase gene were measured in all experiment groups with the CFX Connect Real-Time PCR Detection System. This study shows that *Apis mellifera* L. has a 66.7% mortality rate in mixed *Nosema* infections, a 50% mortality rate in *N. ceranae* infection, a 40% mortality rate in *N. apis* infection, while there is no death in *G. mellonella*. A significant difference was found in the mixed *Nosema* infection group compared to the single *Nosema* infection groups by means of *A. mellifera* and *G. mellonella* (Duncan, *p* < 0.05). *G. mellonella* histopathology also shows that *Nosema* spores multiply in the epithelial cells of greater wax moth without causing any death. The increase in the mRNA level of Phenoloxidase gene in *A. mellifera* was detected (Kruskal–Wallis, *p* < 0.05), while the mRNA level of the Phenoloxidase gene did not change in *G. mellonella* (Kruskal–Wallis, *p* > 0.05). These findings prove that the *Nosema* species can colonize into the greater wax moth, which contributes to the dissemination of these *Nosema* species between beehives.

## 1. Introduction

*Galleria mellonella* L. (Lepidoptera: Galleriidae), the greater wax moth, is known as one of the economic pests in the beekeeping sector, and it can spread all over the world because of its wide range of temperature resistance [1]. This insect species can reproduce simply in both laboratory and its natural environment due to its large size and diet.

This living being can colonize in hives by moving in different ecosystems, and it can transfer diseases, especially honeybee diseases. It was reported that *G. mellonella* could carry and distribute different saprophytic and pathogenic microorganisms [2].

*Nosema* species are obligate intracellular parasites that cause infections called Nosemosis, and honeybee colonies are known to infected with *Nosema apis* and *Nosema ceranae* [3]. An entire honeybee colony, which consists of the queen bee, worker bee and drone, can be infected by these species. Besides honeybees, *G. mellonella* carries *Nosema galleriae* in its intestine as a part of natural microflora [4]. There were also experiments to evaluate the lytic activity through the injection of *Nosema plodiae* and *Nosema algerae* in *G. mellonella*, and these studies mentioned the increase of inhibitory activity of *N. plodiae* and *N. algerae* in *G. mellonella* after injection of these *Nosema* species. [5,6]. Phenoloxidase (PO), which plays a significant role in invertebrates’ immune reaction, is being implicated in the encapsulation of foreign objects through melanization [7]. This defense reaction was observed against fungal infections in insects [8,9]. It is also known that the expression of PO-related genes alters PO activity against infection [10].

Honeybees are the most important creatures among the world, both due to the production of bee products that are crucial in many areas, as well as for maintaining biodiversity by pollination. Unfortunately, large numbers of honeybee colony losses have been observed in many parts of the world in recent years [11,12,13]. The transition of *N. ceranae* to *Apis mellifera* L., and its spread in the period when bee deaths increase, suggests that Nosemosis is the main factor in mass bee deaths [14]. The fact that *Nosema* species have the host-switching feature also supports the idea that it may be effective in mass bee deaths. However, assisting factors in the rapid spread of *Nosema* species among honeybee colonies are unknown. It is considered in the idea that other living beings such as *G. mellonella*, which will accelerate the spread by carrying *Nosema* species between the hives, are effective in this spread as assisting factors. It has already been reported that *G. mellonella* infests weak honeybee colonies exposed to pesticides and diseases, and it is also a threat for healthy colonies [15].

Özgör et al. [15] showed that *N. ceranae* and *N. apis*, which are the most important microsporidia for adult honeybees, are present in the greater wax moth adults and larvae grown in laboratory conditions and collected from apiaries. However, there is no research about the colonization of these two *Nosema* species in the intestine of wax moth and *Nosema*-based time-dependent comparison of wax moth.

This study aimed to prove the possibility of colonization of *N. apis* and *N. ceranae* to the intestine of the greater wax moth, detect the time-dependent differences of the greater wax moth based on the presence of *Nosema* species and examine the effect of *Nosema* species on the level of expression of the PO-related gene in the greater wax moth compared with honeybees.

## 2. Materials and Methods

### 2.1. Selection of Insects

The greater wax moth was reared at the Biotechnology Laboratory (Nicosia, Cyprus) for almost two years. The existence of *Nosema* species was examined experimentally in *G. mellonella* by using the classical *Nosema* detection technique of Cantwell [16], and *N. ceranae*, *N. apis* and *N. galleriae*-free *G. mellonella* late instar larvae were selected for this study. These larvae were allocated to the experimental groups and reared in an appropriate laboratory condition [1]. A temporary condition of collected newly emerged honeybees (*Apis mellifera*) from the sealed brood frame was adjusted by putting it in the cages, which are designed for honeybee experiments, inside the incubator at 34 °C in our laboratory and tested in terms of *Nosema* species by using the same technique. All insects were separated according to the designed experiment.

### 2.2. Preparation of Nosema Stock Solutions

*N. apis* and *N. ceranae* stock solutions were prepared by using infected honeybees, which were previously collected from hives in Cyprus. They are molecularly analyzed in terms of the presence of *Nosema* species, for feeding *G. mellonella* second instar larvae and newly emerged honeybees. *N. apis* and *N. ceranae* positive samples were detected with DNA isolation procedure and Real-time PCR amplification technique by using specific primers for *Nosema* species (*Nosema* spp. specific forward primer ITS-F 5′-TGAATGTGTCCCTGTTCTTTGTAC-3′, *N. apis* specific reverse primer N.apisITS-R 5′-TAATTATAATCTCCTTGGTCCGTG-3′ and *N. ceranae* specific reverse primer NcerITS-R TAAATATAATCTCCTGGTCGGTT) through the modification of the protocol of Bourgeois et al. [17]. Honeybee samples, which are known to carry *N. apis* and *N. ceranae* and stocked at −20 °C for 4 months, were used to obtain feeding stock solution. Thirty honeybees for each *Nosema* species were homogenized separately with 30 mL of distilled water, filtered and centrifuged at 6000 rpm for 10 min to obtain infective spores. After centrifugation, the supernatant was discarded and the pellet used for counting *N.* *apis* and *N. ceranae* spores morphologically on a light microscope with a Neubauer slide. After counting, stock solutions were prepared separately for both *Nosema* species as 10^6^ spores in 100 µL. Stock solutions that were prepared separately for *N. apis* and *N. ceranae* were used to infect insects immediately after processing.

### 2.3. Experimental Infection of G. mellonella and A. mellifera with Nosema spp.

In order to test the infection capability of *Nosema* species in the greater wax moth and to investigate the effect of *Nosema* infection on survival and PO gene expression, four experimental groups were established for each insect species (*G. mellonella* and *A. mellifera*) separately: a group infected with *N. apis*, a group infected with *N. ceranae*, a group infected with mixed *Nosema* species and a control group. The number of individuals in each insect group was determined as 30. All experimental groups were infected simultaneously, using the same batch of *Nosema* stock solutions.

Thirty *G. mellonella* second instar larvae were allocated for each group. The appropriate diet of *G. mellonella* discovered by Chertkova et al. [18] was prepared and the size adjusted to be consumed within 24 h. Each piece of diet was mixed with 100 µL *Nosema* stock solution (10^6^ *Nosema* spores per insect), and it was fed to each *G. mellonella* larva after 24 h of hunger. Experimental wax moth groups were kept in a separate incubator at 27 °C throughout the experiment. Thirty newly emerged worker bees were separated for each group. Each worker bee was fed with 10 µL of a 1:1 ratio of sugar solution diet containing 10^6^ *Nosema* spores on the 1st day of the experiment. Honeybees were anesthetized with CO_2_ to give the full dose to each honeybee. Starved bees were fed a diet containing *Nosema* spores from their mouth with the help of a micropipette until the full dose was consumed [19]. Experimental honeybee groups were kept in a separate incubator at 34 °C throughout the experiment.

Besides, *N. apis* and *N. ceranae* were added to the diet separately, the combination of these two *Nosema* species (mixed infection: 0.5 × 10^6^ *N. apis* spores + 0.5 × 10^6^ *N. ceranae* spores. A total load of *Nosema* spores is 10^6^ per insect, the same as individual infections) was also treated on one group to investigate the synergistic effect of *Nosema* species. Control groups were fed with a *Nosema*-free diet. In the following days, groups continued to be fed with their *Nosema*-free diets. Each group was checked daily, and dead insects removed and counted. Intestines of dead bees and wax moth larvae were individually checked to verify the presence of *Nosema* spores under microscope. Intestines of bees and wax moth larvae were used to check the infection of *Nosema*. Level of infection was determined 6, 9 and 12 days post-infection on thirty moths and bees per sample for each experimental group (*n* = 120 moths and 120 bees) by using the spore count method. The spore count was carried out as in the *Nosema* spore counting method described in the preparation of the *Nosema* stock solution. This experiment was repeated three times.

The *Nosema* infection status was also evaluated with the histopathology of *Galleria mellonella* larvae. The 12th-day larvae after the experimental *Nosema* exposure were used for histopathology. Intestines of frozen larvae were kept in 70%, 80%, 90% and 100% ethanol and xylol series for 1 h for fixation and dehydration. Samples were suspended in paraffin wax for 1 h to complete wax embedding. Samples were cut into 5 μm sections using a microtome and deparaffination done in the incubator for 30 min. Sections were stained with Hematoxylin-Eosin and the samples were examined with a light microscope (Leica D0M 4B) [20].

A separate set of experiments was set up to measure the level of expression of the phenoloxidase-related gene, which determines the effect of *N. apis* and *N. ceranae* on the immune system. Fifteen insects were used for each group. A solution of 10^6^ spores per 10 µL was prepared for each insect’s diet, and the 1st-day feedings of the experimental groups were done as applied to the other groups mentioned above. Considering that the infection cycle of *N. apis* and *N. ceranae* takes a minimum of 96 h from the initial infection of the host cell until new spores are produced [21], for analysis of changes in the level of expression of the PO-related gene after *Nosema* spp. treatment, on the 6th, 9th and 12th days, 5 bees and wax moth larvae from each group were taken and stored at −80 °C after the intestines were obtained by dissection. The study was repeated three times to test the accuracy of the results obtained.

### 2.4. Determination of the mRNA Level of the Phenoloxidase (PO) Gene

Each sample, stored at −80 °C on the 6th, 9th and 12th days after experimental exposure, was used in molecular studies determined by Antúnez et al. [22] for detection of the level of expression of the PO-related gene. The intestine of each stored sample was placed in microcentrifuge tubes with sterile TBS buffer (150 mM NaCl, 50 mM Tris.Cl pH 7.5). The sample was ground in the TBS buffer with a disposable micro-pestle until the intestine was well homogenized into the buffer. Total RNA was isolated from each homogenized sample with the RNA Extraction Kit (Qiagen, Hilden, Germany) by following the manufacturer’s instructions, and then the samples were treated with DNase to remove all DNA from the homogenate. The RNA yield was checked by NanoDrop™ 2000c Spectrophotometer (ThermoFisher, Waltham, MA, USA). All extracted RNA was used to generate the first-strand cDNA by using a M-MLV 1st-strand cDNA synthesis kit (ThermoFisher, Waltham, MA, USA). For each sample, 0.5 ng template RNA and 1 µL primer was used for RT reaction with the final volume of 12 µL [22].

The level of expression of the PO-related gene was detected with Real-time quantitative PCR by using specific primers for Phenol oxidase; PO-F 5′-AATCCATTACCTGAAATTGATGCTTAT-3′, PO-R 5′-TAATCTTCCAACTAATTCATACGCTCTT-3′ were selected for *A. mellifera*; phenoloxidase subunit 1 (LOC113517945), mRNA (NCBI Reference Sequence: XM_026902688.2, 3069 bp) was selected for *G. mellonella* [23,24]. For this analysis, Biospeedy qPCR 2× Master Mix (Bioeksen, Istanbul, Turkey) was selected. Twenty µL final-volume Reaction sample contains 5 µL of 1:10 diluted cDNA, 10 µL of 2× SYBR Green and 0.5 µM of each primer.

Molecular analysis was carried out using a CFX Connect Real-Time PCR Detection System (BioRad, Hercules, CA, USA), and the cycling program of PCR designed to have an initial step at 95 °C for 10 min, elongation step at 95 °C for 10 s (40 cycles) and termination steps at 60 °C for 20 s and 72 °C for 40 s. The specificity of the reaction was checked by analyzing the melting curve, which was obtained through continuous reading over increasing temperatures from 70 °C to 95 °C. The mRNA level of the PO gene was measured in all experiment groups, treated with single and mixed *Nosema* species and in the control group. The level of expression of the PO-related gene was determined by using the mean C_t_ of β-actin as a reference gene [22,25].

### 2.5. Statistical Analysis

The quantitative data from experimental infection analysis were used to compare the colonization and mortality rates between parameters using One-way ANOVA (*p* < 0.05) with a post hoc test (Duncan test). Data on survival were also analyzed with the Kaplan–Meier method, comparing estimated survival functions. Survival curves were compared with a log-rank test (Mantel–Cox). The mean threshold cycle number (C_t_) obtained from molecular studies was used in the statistical analyses (Kruskal–Wallis, *p* < 0.05) to determine the detection of the level of expression of the PO-related gene. For this detection, each PO C_t_ value from Real-time PCR normalized with the mean C_t_ value of the actin.

## 3. Results

### 3.1. Effect of Nosema Infections on Moth and Bee Survival

If we look at the results of both single and mixed *Nosema* infection experiments, no mortality was detected in all experimental groups of *G. mellonella* during the experimental period. *N. apis* and *N. ceranae* have not been found to have a lethal effect on *G. mellonella*.

Cumulative mortality increased over time in all experimental groups but remained lower in the control groups (~10% mortality rate) in *A. mellifera*. A statistically significant difference was found between the experimental groups (One-way ANOVA, *p* < 0.05). All three experimental groups exhibited significantly higher mortality rates than the control group. Although single *Nosema*-infected bee mortality was not statistically different (mortality rates: 40% for *N. apis*, 50% for *N. ceranae*; *p* > 0.05), mixed *Nosema* infection (*N. apis* and *N. ceranae*) was found to have a significant difference compared to other groups with the highest mortality rate (66.7% mortality rate; Duncan, *p* < 0.05). In the 12-day experimental infection, when the mortality comparison was compared between the experimental groups, the cumulative mortality increased at the same rates in all groups except the control group. Although the increase in cumulative mortality is seen to be a significant difference between days, especially six days post-infection, daily deaths were observed at the same rates in all groups (One-way ANOVA, *p* < 0.05).

**Figure 1:**
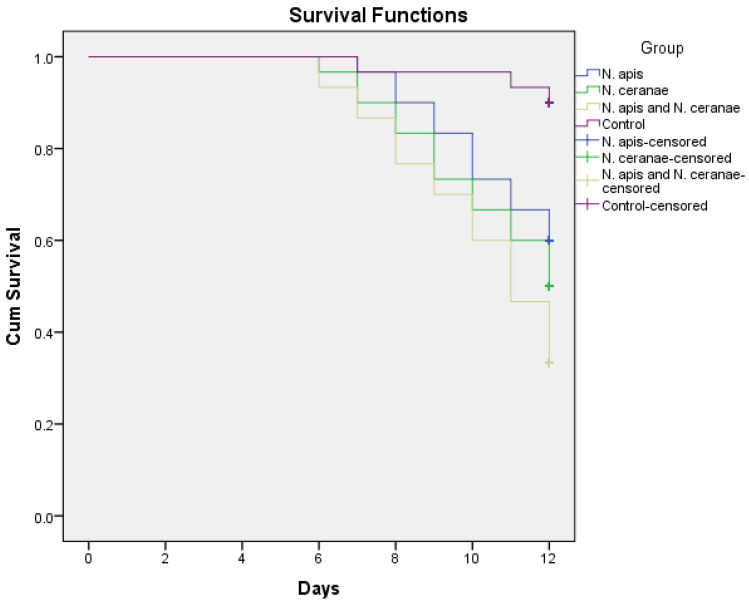
Cumulative survival of newly emerged worker honeybees inoculated with 10^6^ *Nosema* spores/bee (single and mixed infection) or not inoculated (control group). Survival functions were estimated with the Kaplan–Meier method.

Bees inoculated with single and mixed *Nosema* spores had significantly lower survivorship rates than control bees (Log-rank test, χ^2^ = 20.214, *p* < 0.01; Figure 1). When the single infections with *N. apis* and *N. ceranae* were evaluated, there was no significant difference between the survival rates (χ^2^ = 0.693, *p* > 0.01), whereas, in the case of mixed *Nosema* infection, the survival rate was found to be significantly reduced in honeybees compared to single *Nosema* infections (χ^2^ = 6.285, *p* < 0.01). These results demonstrate that mixed *Nosema* infection is more pathogenic to honeybees than single *Nosema* infection and can impact the population growth of colonies.

### 3.2. Infection Capability of Nosema Species to Moth and Bee

**Figure 2:**
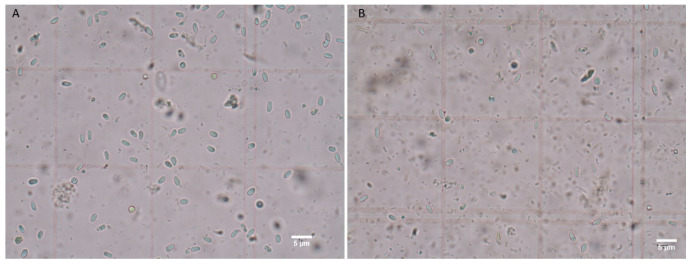
Microscopic images of *Nosema* spores in the intestine of Apis mellifera worker bees (**A**) and *Galleria mellonella* larvae (**B**). 12 days after mixed *Nosema* exposure. Figure scales are 5 μm.

**Figure 3:**
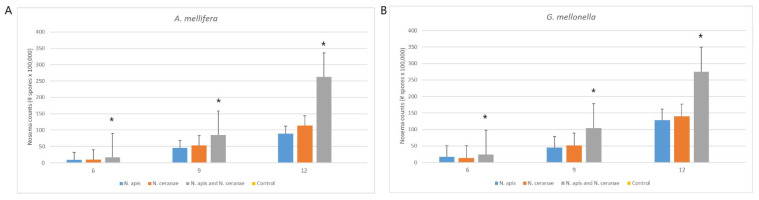
Level of *Nosema* infection (spore load) in *A. mellifera* (**A**) and *G. mellonella* (**B**) fed with single and mixed *Nosema* spp. (*N. apis* and *N. ceranae*) compared with the control group. Level of infection was determined 6, 9 and 12 days post-infection on thirty moths and bees per sample for each experimental group (*n* = 120 moths and 120 bees). Data show mean ± SE. Asterisks indicate values significantly different within the groups (Duncan, *p* < 0.05).

The infection status of *N. apis* and *N. ceranae* spores and colonization of these spores into *G. mellonella* and *A. mellifera* were evaluated as a result of the ingestion to the digestive system by diet (Figure 2). The existence of *Nosema* spores was not detected in the control groups of *G. mellonella* and *A. mellifera*. However, the level of *Nosema* infection (spore load) was significantly different between groups infected with mixed *Nosema* infection (*N. apis* and *N. ceranae*) and groups infected with single *Nosema* species in both bees and wax moth larvae (Duncan, *p* < 0.05). Although the infection of *N. ceranae* spores was lower compared to *N. apis* in both insect species, there was no statistically significant difference between these two groups (Duncan, *p* > 0.05) (Figure 3).

Infection capability of *Nosema* species was determined on the 6th, 9th and 12th days after infection in both *A. mellifera* and *G. mellonella* experimental groups. A significant difference was found between the infection capabilities determined depending on the days in all experimental groups of *A. mellifera* and *G. mellonella* except the control group (One-way ANOVA, F = 9.966, *p* < 0.05; F = 11.876, *p* < 0.05 respectively). Interestingly, at twelve days post-infection, all infected groups had a significantly higher number of spores than six and nine days post-infection because of the slight activation of spores (Figure 3).

In addition to the single and mixed infectious states of *Nosema* species, infection capabilities between *A. mellifera* and *G. mellonella* were also statistically compared. It was found that there was no significant difference between *A. mellifera* and *G. mellonella* in terms of *Nosema* spore counts in all experimental groups in the six, nine and twelve days post-infection (One-way ANOVA, F = 2.247, *p* = 0.148; F = 0.087, *p* = 0.771; F = 0.225, *p* = 0.640 respectively).

**Figure 4:**
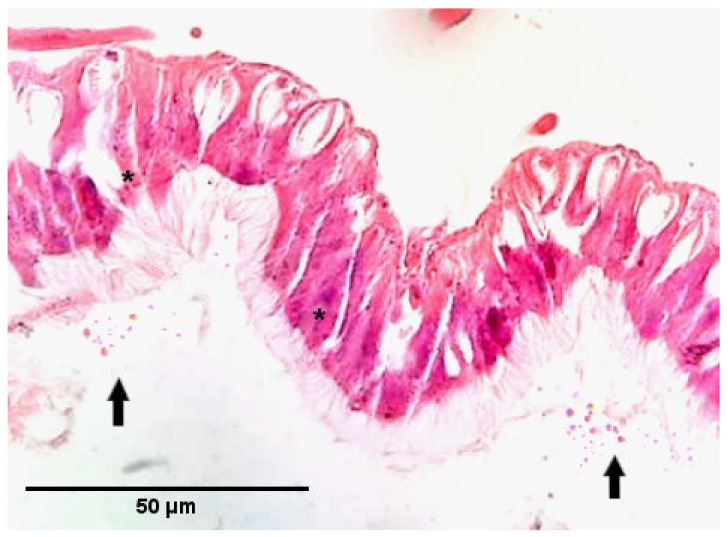
Histological image of the midgut of the greater wax moth larvae. Cylindrical epithelial cells with microvilli (asterisks) infected with 10^6^ *Nosema* spores (infection with mixed *Nosema* spores (*N. apis* and *N. ceranae*), 12 days post-infection). Among the epithelial cells, goblet cells with a normal structure are also seen. There are also mature *Nosema* spores (arrows) in the lumen. Hematoxylin-eosin staining.

The histopathology result of Galleria mellonella larvae also showed the *Nosema* spore germination and multiplication inside the epithelial cells of the midgut (Figure 4).

### 3.3. Effect of Nosema Infections on the Level of Expression of the PO-related Gene in Moth and Bee

**Figure 5:**
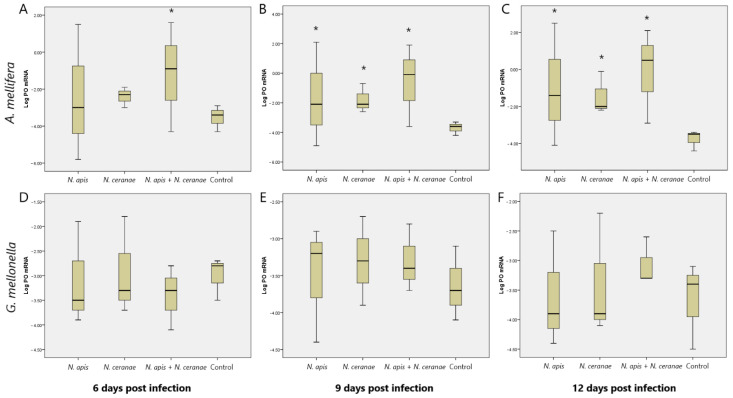
Effect of time-dependent *N. apis* and *N. ceranae* infection on the expression of Phenoloxidase-related gene in *A. mellifera* at 6, 9 and 12 days post-infection (**A**–**C**), respectively) and *G. mellonella* at 6, 9 and 12 days post-infections (**D**–**F**), respectively) (box plots graphics). Asterisks indicate statistically significant differences (Kruskal–Wallis, *p* < 0.05).

The mRNA expression level of the PO gene was determined 6, 9 and 12 days after experimental infection. PO-specific amplification was confirmed for the primers of PO in the melting curve analysis and through the Tm values. mRNA levels of PO increased significantly at the mixed infection of *N. apis* and *N. ceranae* in *A. mellifera* compared to control or single-*Nosema*-infected bees on the 6th day post-infection (Kruskal–Wallis, *p* < 0.05; Figure 5A). Besides, the expression of this gene increased in all *Nosema* infection groups compared to the control group at 9 and 12 days post-infection (Kruskal–Wallis, *p* > 0.05; Figure 5B,C). In *G. mellonella*, there was no increase in mRNA levels of PO in both single and mixed *N. apis* and *N. ceranae* infection groups compared to the control group at 6, 9 and 12 days post-infection, and no statistical difference was found among all experimental groups (Kruskal–Wallis, *p* > 0.05; Figure 5D–F). No decrease was observed in any infection in both *A. mellifera* and *G. mellonella* compared to the control group.

## 4. Discussion

Most *Nosema* species have a wide range of potential hosts from Lepidoptera to Hymenoptera [26]. Some insects may be their natural hosts, while they may show parasitic effects on others. This shows that most *Nosema* species do not show host specificity and have the host-switching feature. In addition to *N. apis*, which has existed in European honeybees (*Apis mellifera*) for many years, *N. ceranae*, which has been seen in Eastern honeybees (*Apis cerana*) in recent years, has switched its host. Massive bee deaths in *A. mellifera* colonies are thought to have become widespread since 2006 due to *N. ceranae* [14,27]. It is considered that different pests which interact with bees, as well as physical factors, may be effective in the spread of *N. ceranae* in *A. mellifera* by carrying this microsporidian.

In the experimental infection, it was determined that there was no death in the greater wax moth during the experiment. All *G. mellonella* experimental groups, which were fed with *N. apis* and *N. ceranae* species, have parasitic effects on honeybees in their natural environment and continued to live at the end of the experiment. It is thought that infection with *Nosema* species does not cause a fatal effect on *G. mellonella* since there are *Nosema* species detected in *G. mellonella*, and no death was found before in the experimental studies done. No death was observed after an experimental infection of *G. mellonella* with *Nosema pyrausta* in the study conducted by Tokarev et al. [28]. Hence, it was hypothesized that *G. mellonella* is a resistant model host for *Nosema* species. The results obtained in our study fully support this hypothesis.

Our results indicate that single and mixed *Nosema* infections increase the mortality and reduce the survivorship in *A. mellifera* (Figure 1). The cumulative mortality caused by *N. ceranae* was not significantly higher than the mortality induced by *N. apis*. The effects of single *Nosema* infections on survival rates were also almost the same. Similarly, Forsgren and Fries [3] found that *Nosema* species showed similar virulence by comparing the effect of *N. apis* and *N. ceranae* on bee mortality. In contrast, Martín-Hernández et al. [29] indicated that *N. ceranae* had a high virulence and increased mortality compared to *N. apis* in the experimental infection studies related to energy stress. Furthermore, Paxton et al. [14] have shown that *N. ceranae* induced significantly higher mortality than *N. apis* in their cage experiments with honeybees. This study obtained different results according to our study, in which we conducted similar studies with the *Nosema* dose (10^6^) and the cage experiment. This suggests that the effect of *Nosema* species on the regional diversity and subspecies of the European honeybee can vary, or the defense system in the immune system of honeybees has started to strengthen because *N. ceranae* has long been in European honeybees. Besides, the fact that the mixed *Nosema* infection caused a significant increase in bee mortality compared to single *Nosema* infection shows that the mortality rate increased in the case of co-infection in European honeybees, which has been frequently encountered in recent years. In the study of Williams and colleagues [30], honeybee mortality was significantly higher with *N. ceranae* than *N. apis* or mixed infections, and mixed infection resulted in mortality similar to *N. apis* because of interspecific competition. In our study, the significant increase in bee mortality in mixed infection compared to single *Nosema* infection can be explained by the fact that *N. ceranae* naturally increases the co-infection with *N. apis* and increases bee deaths by decreasing intra-host competition.

These are the first data on experimental infection of the greater wax moth with the microsporidia *N. apis* and *N. ceranae*; besides, the infectivity of *Nosema* species to *A. mellifera* was previously mentioned [14,29,31]. Our results show that these *Nosema* species develop well in both *G. mellonella* and *A. mellifera* and that their infectivity starts within 6 days by completing their intracellular life cycle (Figure 2). *Nosema* spp. were not detected in the control group among the greater wax moth experimental infection groups, and a significant difference was found in the mixed *Nosema* infection group compared to the single *Nosema* infection groups in all evaluated days (Figure 3). The absence of a difference between *N. apis* and *N. ceranae* individual experimental groups indicates that these *Nosema* species have approximately similar sporulation properties in the digestive system of *G. mellonella*. Injection assays related to *Nosema* species showed that *Nosema* spp. appear to have either a consistent level of infection or no infection in the greater wax moth. In this context, it has been shown that there is no death in greater wax moth larvae treated with *Nosema pyrausta* [28], *Nosema furnacalis* [32] and *Nosema bombycis* [33]; they have high resistance to these species and can carry these species in their midgut with consistent levels. In this study, a consistent increase in the level of infection in all groups without mortality was documented, and similar results were obtained. On the other hand, Malysh et al. [34] demonstrated that *N. ceranae* isolated from *A. mellifera* infects the beet webworm *Loxostege sticticalis* L., another lepidopteran host, without any damage both in laboratory conditions and the natural environment. However, the results of this study of Preston et al. [35] showed that *Nosema maddoxi* is widely distributed throughout a non-lepidopteran host, *Halyomorpha halys* (Hemiptera: Pentatomidae) populations in the USA, and impact *H. halys* population dynamics. This is further proof that the species in the order Lepidoptera, including the greater wax moth, have high resistance to *Nosema* species in all conditions.

Experimental infection of *A. mellifera* gave similar results to the results of *G. mellonella* (Figure 3). The *Nosema* spore counts in *A. mellifera* were significantly higher in mixed *Nosema* infection than in single *Nosema* infections, while there was no *Nosema* infection in the control group. Milbrath et al. [36] found that *A. mellifera* with mixed infections of both *N. apis* and *N. ceranae* has higher spore loads than single infections, and *N. ceranae* does not have a strong within-host advantage for spore growth and infectivity compared with *N. apis*. This study showed very similar results to our study, revealing that *N. ceranae* does not have a more dominant infective feature than *N. apis*. Besides, similar results were obtained in both honeybees and greater wax moths in terms of infectivity, but the absence of death in the moth, unlike the bees, confirms that the greater wax moth is a resistant living being against *Nosema* species. Moreover, Gisder et al. [37] demonstrated that there is no infection and production of *Nosema* spores in bumblebees (*Bombus terrestris*) ingested with *N. ceranae* spores. This situation shows us that *Nosema* infection will not develop in all species under the order Hymenoptera, and it can be seen in certain species, which is common in species under the genus Apis.

This study further examined the effects of 10^6^ *Nosema* spores (infection with mixed *Nosema* spores, 12 days post-infection) on *G. mellonella* larvae using wax (Hematoxylin-eosin) histology (Figure 4). *Nosema*-infected epithelial cells with different intracellular parasite stages are clearly seen in this wax section. Moreover, it has been determined that mature *Nosema* spores are found in bulk in certain parts of the lumen. This is one of the pieces of evidence that *Nosema* spores can infect the greater wax moth and do not cause any death in the greater wax moth while multiplying inside the epithelial cells.

Phenoloxidase (PO) is an increased enzyme against pathogens such as bacteria, fungi and parasites in both *A. mellifera* and *G. mellonella* [10,22]. Although PO is just one of the factors related to immunity, it contributes to the antibacterial properties of honeybee hemolymph [38]. After the treatment with the pathogen, the mRNA expression level of the Phenoloxidase gene was measured periodically, and the possible state of the immune system against the pathogen was evaluated (Figure 5). In this study, the significant increase in the mRNA level of PO in the mixed *Nosema* infection on the 6th day, and in all infection groups except the control group after the 9th day, shows that *N. apis* and *N. ceranae* in the honeybees stimulate the immune system by causing a pathogenic effect. Antúnez et al. [22] showed that 7 days post-infection with *N. apis* induced higher mRNA expression of the PO gene than *N. ceranae* in *A. mellifera*. However, Alaux et al. [39] found that PO activity was neither up- nor down-regulated by the *Nosema* challenge at days 5 and 10. They emphasized that the lack of immune response might be explained by deficient immunoregulatory activation or inactivation of spores in this study. The results of these studies are compatible with our results and that *Nosema* infection in bees provided activation of the expression of PO-related gene. Also, it was found for the first time that mixed *Nosema* infection in honeybees increased the PO gene level compared to single *Nosema* infection in this study.

The PO level normally increases in the presence of infections in the activation of the immune system in the greater wax moth as in other insect species. The absence of a significant difference in the mRNA level of PO in all *Nosema* experimental groups compared to the control group indicates that *N. apis* and *N ceranae* did not activate the expression of the PO-related gene in the greater wax moth. Although *Nosema* spore count increases timely in the greater wax moth in the *Nosema* experimental infection as in honeybees, the absence of death in moth during the experiment strongly supports that *N. apis* and *N. ceranae* are not perceived as pathogens by the immune system of *G. mellonella* and colonized into this living being. However, in some cases, there are also studies with pathogens such as *Xenorhabdus nematophilus* [40] and *Aspergillus oryzae* [41], which reduce the level of PO by immunosuppression. Contrary to this study, the fact that there was no change in PO level in our study, as well as no death, indicates that *N. apis* and *N. ceranae* are not pathogens for the greater wax moth.

## 5. Conclusions

The single and mixed virulence effect of *Nosema* species may vary depending on the host species and environmental conditions. *A. mellifera* has some mortality rates in mixed and single *Nosema* infection cases, while the absence of death in *G. mellonella* shows that the greater wax moth has a resistant structure against *Nosema* species. However, the fact that all infection groups of *N. apis* and *N. ceranae* species did not show a significant difference between *A. mellifera* and *G. mellonella* demonstrates that these two species could be colonized without causing death of the greater wax moth. Despite the increase in the mRNA level of PO in *A. mellifera*, the immune system in *G. mellonella* remained inactive, and the mRNA level of the PO did not change. This proves that the infection remains stable in this organism, and *N. apis* and *N. ceranae* can colonize into the midgut of the greater wax moth like *N. galleriae*. This study also shows that *G. mellonella*, which is one of the natural pests of honeybees, carries *N. apis* and *N. ceranae* species between hives without any symptoms and increases its spread.

## Data Availability

Not applicable.
